# A formalized description of the standard human variant nomenclature in Extended Backus-Naur Form

**DOI:** 10.1186/1471-2105-12-S4-S5

**Published:** 2011-07-05

**Authors:** Jeroen F J Laros, André Blavier, Johan T den Dunnen, Peter E M Taschner

**Affiliations:** 1 Department of Human Genetics, Center for Human and Clinical Genetics, Leiden University Medical Center, Leiden, the Netherlands; 2 Interactive Biosoftware, Rouen, France

## Abstract

**Background:**

The use of a standard human sequence variant nomenclature is advocated by the Human Genome Variation Society in order to unambiguously describe genetic variants in databases and literature. There is a clear need for tools that allow the mining of data about human sequence variants and their functional consequences from databases and literature. Existing text mining focuses on the recognition of protein variants and their effects. The recognition of variants at the DNA and RNA levels is essential for dissemination of variant data for diagnostic purposes. Development of new tools is hampered by the complexity of the current nomenclature, which requires processing at the character level to recognize the specific syntactic constructs used in variant descriptions.

**Results:**

We approached the gene variant nomenclature as a scientific sublanguage and created two formal descriptions of the syntax in Extended Backus-Naur Form: one at the DNA-RNA level and one at the protein level. To ensure compatibility to older versions of the human sequence variant nomenclature, previously recommended variant description formats have been included. The first grammar versions were designed to help build variant description handling in the Alamut mutation interpretation software. The DNA and RNA level descriptions were then updated and used to construct the context-free parser of the Mutalyzer 2 sequence variant nomenclature checker, which has already been used to check more than one million variant descriptions.

**Conclusions:**

The Extended Backus-Naur Form provided an overview of the full complexity of the syntax of the sequence variant nomenclature, which remained hidden in the textual format and the division of the recommendations across the DNA, RNA and protein sections of the Human Genome Variation Society nomenclature website (http://www.hgvs.org/mutnomen/). This insight into the syntax of the nomenclature could be used to design detailed and clear rules for software development. The Mutalyzer 2 parser demonstrated that it facilitated decomposition of complex variant descriptions into their individual parts. The Extended Backus-Naur Form or parts of it can be used or modified by adding rules, allowing the development of specific sequence variant text mining tools and other programs, which can generate or handle sequence variant descriptions.

## Background

Unambiguous descriptions of genetic variants are important to prevent mistakes in the clinical diagnosis of disease [1]. The Human Genome Variation Society (HGVS) promotes the use of a standard human sequence variant nomenclature, which has gradually evolved as the result of continuous additions and changes [1-4]. The standard nomenclature has been designed mainly for use in tables in the literature and in gene variant databases (locus-specific mutation databases, LSDBs). Due to technological improvements (next generation sequencing, microarrays), a growing number of complex variants are now detected at relatively high resolution, bridging the traditional divide between chromosome analysis in cytogenetic diagnostics and single gene analysis in DNA diagnostics. This is also reflected in recently proposed extensions of the standard nomenclature [5] and the incorporation of symbols from the International System for Human Cytogenetics Nomenclature (ISCN 2009) [6].

These extensions increase the complexity of the standard nomenclature, which is becoming more difficult for non-experts to understand and use. To assist clinicians and researchers, computational tools, such as the Mutalyzer sequence variant nomenclature checker [7], have been developed. LSDB curators using LOVD software [8] can improve the quality of newly submitted variant descriptions via the integrated Mutalyzer module.

Curators contributing the majority of variant data themselves would welcome automated extraction of variant information at the DNA, RNA and protein levels to cope with increasing amounts of data in the literature. The text mining community has started the BioCreative initiative [9] to assess newly developed methods for the efficient extraction of data. Several tools have been developed to extract variant data from the literature (See [10,11], references therein and other articles in this special issue). Current efforts have mainly been focussing on the protein level [10], which reduced the complexity of the search patterns and classifiers needed for these methods. When users have downloaded literature relevant for their genes of interest, stand-alone programs (e.g. MutationFinder [12], mSTRAP [13]) could be used to extract variant information from selected texts. On-line databases storing variant data obtained by text mining tools (e.g. GoGene [14], OSIRIS [15]) could be queried directly for gene-specific information. This approach would be more convenient for curators, so we compared the contents of these databases with those of the corresponding LSDB for the SDHD gene. This raised the questions whether the complexity of the descriptions at the DNA and RNA levels prohibited their automated extraction by text mining tools and how this might be solved.

The textual nomenclature recommendations on the HGVS website are spread over different pages with relatively simple examples, making it difficult to get a complete overview of the complexity of sequence variant description constructs up to the genotype level. The HGVS nomenclature can be regarded as a scientific sublanguage [16] using specific typographic and orthographic conventions to communicate information about sequence variants. A formal description that aids in understanding the structure of the nomenclature is currently lacking. Here we present two formal descriptions of the syntax in Extended Backus-Naur Form (EBNF) [17]: one at the DNA-RNA level and one at the protein level. The DNA-RNA level EBNF has been used to construct the Mutalyzer 2 parser and could form the basis for other tools. Parts of the EBNF could easily be extended to accommodate the wider variability of descriptions encountered in the general literature or used separately to generate parsers for sequence variant text mining.

## Methods

### Standard human sequence variant nomenclature

Version 2.0 of the HGVS standard sequence variant nomenclature [1-4] was used for the Extended Backus-Naur Form. The consistency of the nomenclature was checked, leading to removal of symbol redundancy and clarification of specific rules after communication with the HGVS (den Dunnen, manuscript in preparation). These changes and extensions, which have not yet been formally approved by the HGVS, have been incorporated in the formal descriptions presented here. The extensions included the following:

i) The nested change format, which supports descriptions of complex changes such as structural variants [5]. In this format, an A to C substitution at position 158 within an inverted region (position 100 to 200) could be described at the genomic DNA level as an inversion with the substitution as a sub-allele shown between curly braces, e.g. g.100_200inv{158A>C}. In contrast, the conventional description would describe it as the deletion-insertion g.100_200delinsAB23456.7 using the Genbank accession number of the sequence of the inverted region with the substitution.

ii) The symbols / (slash) and // (double slash) used in the ISCN2009 cytogenetic nomenclature to describe mosaicism and chimerism, respectively [6]. These symbols would separate the variants found in the different cell lineages, e.g. g.[158A>C/124C>T] for two different substitutions found in two different cell lineages of a individual with germline mosaicism.

iii) The description format used with the new Locus Reference Genomic (LRG) reference sequences [18]. The fixed annotation of LRG sequences might contain information about more than one transcript and protein from the same gene. For descriptions at the RNA and protein level, these have to be specified using suffices, e.g. LRG_1t1 and LRG_1p1. New symbols pending HGVS approval are explained in Table 1.

**Table 1:** New symbols and symbol applications in the extended standard human sequence variant nomenclature^a^

d	Downstream. Position number prefix for coding DNA positions following the end (3') of the transcript. Example: c.*405+d256G>T
n	Position number prefix for non-coding DNA positions. Numbering starts at the first nucleotide of the non-coding transcript. Example: n.46G>T

p	Suffix to specify protein isoforms in descriptions using LRG sequences (Locus Reference Genomic) [18]. Example: LRG_1p1

t	Suffix to specify transcript variants in descriptions using LRG sequences. Example: LRG_1t1

u	Upstream. Position number prefix for coding DNA positions upstream (5') of the start of the transcript. Example: c.-110-u256G>T

_i	Gene symbol suffix to specify protein isoforms in protein variant descriptions using genomic reference sequences. Example: DMD_i2

_v	Gene symbol suffix to specify transcript variants in coding DNA variant descriptions using genomic reference sequences. Example: DMD_v2

^	Exclusive or: to combine DNA descriptions, which are derived from protein level descriptions. Example: backtranslation of p.Ser124Arg, where the Ser-124 codon at c.370_372 is AGC. The variant should be described as c.[370A>C^372C>R] to reflect that arginine can be encoded by six possible codons, AGR (AGC and AGT) and CGN (CGA, CGC, CGG and CGT), respectively.

/	Allele separator in mosaic cases. Used in ISCN [5]. Example: c.[=/85C>T]

//	Allele separator in chimaeric cases. Used in ISCN [5]. Example: c.[=//85C>T]

{ }	Curly braces enclose "sub-alleles", changes within the range of duplications, inversions, gene conversions and insertions. Example: c.24_65inv{46G>T} (See [4,5] for details)

;	Replaces + in SingleAlleleVarSet, MultiAlleleVars and MultiTranscriptVar

(;)	Replaces (+) indicating uncertain phase in UnkAlleleVars. In general, parentheses are used to indicate uncertainty.

^a^ See http://www.hgvs.org/mutnomen for a full list of symbols and their use.

### Development of nomenclature grammar using Extended Backus-Naur Form

We have used a top-down approach to manually build a set of syntactic and lexical rules out of the textual nomenclature recommendations on the HGVS website [2]. In EBNF, the grammar of a language is defined by a set of rules like the following: Sentence → subject verb complement '.' Rules are conventionally represented using an arrow separating the head and the body. The rule above says that a sentence is made of a subject, a verb and a complement, and is terminated by a '.' character, in that order. Each rule has a nonterminal symbol in its *head* (left-hand side) and a *body* defining how terminal and nonterminal symbols link together. Sentence and subject are non-terminal symbols, i.e. they must be defined by a rule. '.' is a terminal symbol and requires no further definition. In addition to simple sequences of symbols and characters in the right-hand part, like in the above rule, a few notational conventions enable the description of more complex constructs:

• A set of literals (characters) is described like this: [A-Z], which defines the set of latin uppercase letters.

• Alternatives are denoted by |, as in: Nucleotide → 'A' | 'C | 'G' | 'T'

• Optional constructs are denoted by ?, as in: sentence → subject verb complement? '.' meaning that a correct sentence can have a complement, which is optional.

• Zero or more occurrences of a construct are denoted by *. One or more occurrences of a construct are denoted by ^+^:

Number → [0-9]^+^ defines numbers as a series of 1 or more digits.

The EBNF combines *terminal symbols* (letters, digits, and other typographic characters) and *nonterminal symbols* in a recursive set of rules defining a language. Since variation descriptions at the DNA and RNA levels share many features, both conceptually and syntactically, we have unified them into one set of EBNF rules, while a separate grammar has been derived for the protein level. Terminal symbols including all characters used in the HGVS standard sequence variant nomenclature [2] were depicted using **bold** print in Additional Files 1 and 2. For example, the description of a deletion of amino acids at the protein level was expressed by the following rule:

Del → AALoc **'del'**

The head, Del, was the nonterminal symbol defined by this rule. The body combined another nonterminal, AALoc, which is defined by another rule, and the terminal **'del'.** This rule stated that a protein-level deletion must be described as the name of an amino acid along with its position (AALoc), followed by the three letters 'del', as in Glyl23del.

### Mutalyzer 2 parser construction

The Mutalyzer 1.0.4 Name Checker used regular expressions to parse relatively simple variant descriptions before checking their correctness [7]. Updating and testing these regular expressions containing many escape characters due to all symbols used in the nomenclature was difficult and time consuming, but necessary to include every nomenclature change. Reliable recognition of allele and genotype descriptions required designing regular expressions with more complexity. Therefore, we decided to simplify parser maintenance using the approach described below. The new nested change format introduced an additional description complexity, which made parsing with regular expressions impossible. The Pyparsing package [19] is an object-oriented tool kit for building *recursive descent* parsers. This type of parsers has a structure resembling that of the grammar recognized and supports a recursive set of rules. We used Pyparsing to transform the EBNF of the HGVS standard sequence variant nomenclature at the DNA and RNA levels into the Python code that forms the parser. Since no parser generation step is involved, the source code of the parser contained the original description of the nomenclature in a human-readable format. In this EBNF-like Python code, we have indicated that a named object must be formed when a particular rule is applied. For example, when a (position) range is parsed, the rule that defines a range is applied and an object containing two positions must be formed. By selectively assigning objects to rules, we generated a nested object: the Mutalyzer 2 parse tree.

## Results

### Formal description of DNA and RNA variant nomenclature

New symbols have been introduced by recent changes and extensions of the sequence variant nomenclature pending formal approval by the HGVS (Table 1). These symbols and extensions have been included in the DNA and RNA variant nomenclature grammar represented in EBNF in Additional File 1. The top level rule, the starting point from which the highest level of complexity can be processed using the EBNF, is named "Var". In principle, this production rule supports detailed descriptions of complete genotypes, even for individuals with mosaicism and chimerism. It also refers to "SingleVar", the production rule for the simplest variant form, which handles the format <Reference sequence.version_number>:<variant_description> (e.g. NM_003002.2:c.274G>T for the substitution of G at coding sequence oriented position 274 by T in reference sequence NM_003002.2). This format contains the minimal information necessary to reliably generate the variant sequence by hand or with the Mutalyzer Name Checker [7]. In the literature and LSDBs, the complete format is rarely used. The reference sequence should be mentioned in the Materials and Methods section or in the table legends, but is frequently lacking. In LSDBs, it should be mentioned on the gene information page. Variants in tables are commonly described using the variant_description part. The Mutalyzer module integrated in LOVD combines the reference sequence accession number with the variant_description part to generate the complete format to check the description.

For backwards compatibility, rules for several description types used in previous versions have been listed. These "deviation rules" include "IVS" (Intervening Sequence) followed by intron number, which has been used to specify intronic positions. Although often causing confusion, the description "EX" (exon) followed by exon number has been included, because of its frequent use in the Online Mendelian Inheritance in Man (OMIM) database [20].

### Formal description of protein variant nomenclature

Although formal experimental proof of changes at protein level are mostly lacking, it is common use to report the predicted effects from the variants detected at the DNA level. The protein variant grammar represented in EBNF follows the current version of the standard protein variant nomenclature (Additional File 2). The top level rule, the starting point from which the highest level of complexity can be processed using the EBNF, is named "ProteinVar". The production rule for the simplest variant form, "SingleVar", consists of the reference sequence and the variant description parts. The EBNF includes rules for the preferred three-letter amino acid code as well as the one-letter amino acid code.

### Variant description handling in the Alamut software

The grammars were initially created to help implement the variant parsing and generating capabilities of the Alamut mutation interpretation software [21]. Alamut is a decision-support system dedicated to variant interpretation in human genetics. It integrates several molecular and clinical data sources along with missense and splicing prediction tools inside a graphical gene browser. Users can enter variants manually, by selecting affected nucleotides graphically and specifying the type of the change. The software can also import variants from text files holding descriptions based on the nomenclature. Variant parsing in Alamut uses regular expressions derived from a subset of the DNA-level grammar defined here, since the software currently handles only substitutions, insertions, duplications, deletions, and insertion-deletions. The grammar also serves as an implementation guide for generating conformant descriptions out of user-entered variations. The DNA and protein-level grammars were also used to implement the text mining tool provided with Alamut. However, they had to be extended so as to be able to cope with the numerous lexical variations found in the literature. For instance, almost 10% of DNA variant descriptions detected in PubMed abstracts use '/', '->', or '-->' instead of '>' for substitutions.

### Construction of a context-free nomenclature parser

We successfully incorporated the DNA and RNA variant nomenclature grammar represented in EBNF into a recursive descent parser in version 2.0 of the Mutalyzer software suite [7]. In the Syntax Checker, which precedes the Mutalyzer 2 nomenclature checker, the parser is used as an acceptor. If a variant description is not syntactically correct, this interface will return an error message, indicating the position in the variant description where the parser halted. This interface can be used to rapidly check the syntax of many variants (e.g., in a database) even when a reference sequence is not known or available. The implementation of the Mutalyzer 2 Syntax Checker can handle separate descriptions in the standard variant nomenclature. Although not intended to support full-text analysis, the batch mode of the Syntax Checker could be used to recognize (parts of) HGVS-compliant descriptions (e.g. NM_003002.2:c.274G>T, c.274G>T and 274G>T) from a tab delimited text file containing a single word on every line [22]. Descriptions of single variants without nesting including the reference sequence accession number are recognized or rejected equally well by both Mutalyzer versions (results not shown). More complex allele descriptions with nested changes can only be parsed correctly by Mutalyzer 2.

### Variant data collection and text mining tools

While most LSDBs use specific software [8] for direct submission of new variants data, most data are still manually extracted from the literature and entered manually by LSDB curators. We have investigated if LSDB curators could use the on-line databases GoGene [14] and OSIRIS [15], which are filled using text mining tools, as a source of variant data. For an example of the efficiency of these tools, we reviewed variant data for the SDHD gene, for which GoGene (visited Dec 3, 2010) lists a maximum of 30 publications describing a total of 6 nonsense and 15 missense variants at the protein level. OSIRIS (visited Dec 3, 2010) contains 1 silent, 3 missense and 39 non-coding variants described at the DNA level. In contrast, the SDHD LSDB [23] (visited Dec 3, 2010) contains 253 variants of which 24 have been submitted directly. The other variants were extracted manually by the curators from papers obtained by regular literature searches. The 122 unique variants include 54 coding region substitutions and 12 intronic substitutions at the DNA level. At the protein level, 27 missense and 19 nonsense variants are listed.

## Discussion

The grammars proposed here have concentrated the lexical items and syntactic constructs which are scattered throughout the HGVS nomenclature web pages in one place. This obviously helps finding potential problems or ambiguities and adding new constructs. This first effort to formalize variant nomenclature may eventually lead to an integrated grammar. The close similarity of the grammar of RNA and DNA variant descriptions already allowed us to combine these variant nomenclature sublanguages. A separate grammar for the protein variant nomenclature sublanguage had to be designed, since protein variant descriptions are quite different from DNA and RNA level descriptions. To keep pace with the evolution of the standard nomenclature, updated versions of the EBNFs will be regularly available from the Mutalyzer website [22]. The EBNFs presented here could serve several purposes: as a reference for reasoning about the nomenclature (e.g. identifying constructs which are allowed by the current nomenclature grammar, but invalid), for further additions (e.g. deviation rules to catch incorrect descriptions), and for implementation into software (e.g. generators of correct variant descriptions, variant callers for next generation sequencing data, text mining tools). We consider the implementation of DNA and RNA level EBNF as a step towards the production of correct variant descriptions. The sublanguages defined here are supersets of the actual nomenclature, which are not restricted by the semantic rules of the standard human sequence variant nomenclature [2]. The most important semantic rules, which cannot be described in EBNF format, have been recapitulated [5]. Therefore, implementations based on the grammars should restrict the supersets of otherwise syntactically correct constructs by semantic checking. Future work will include the addition of constraints to prevent invalid constructs (e.g. AA1_AA3 for a protein range in mixed single-letter and three-letter amino acid code).

Currently, a gap exists between the increasing amounts of variant data generated with next generation sequencing and array technologies and the tools available to extract DNA variant descriptions and their functional consequences at the RNA and protein levels automatically from textual or tabular formats. Traditionally, the focus of text mining tools was on the functional consequences of amino acid substitutions in relation to protein engineering and biotechnology. Our SDFID data comparison confirmed that efforts to extract variant data are currently limited to the protein level in GoGene and mainly restricted to entries with dbSNP identifiers in OSIRIS. Both data sets were smaller than that contained in the corresponding gene variant database, which was manually filled by curators (90 %) and submitters (10 %). One of the explanations might be that insufficient insight into the complex structure of the sequence variant nomenclature and the format of variant descriptions at the DNA and RNA level was unclear. Another explanation might be that it is difficult for text mining tools to identify the reference sequence in the Materials and Methods section or in the table legends. Both make reliable extraction of variant data more difficult. Most LSDB curators would prefer on-line tools which automatically send updates with extracted variants and can be queried on a regular basis, rather than stand-alone programs, which will have to be separately installed and may require IT assistance. Therefore, a web-based implementation of MutationFinder, mSTRAP or similar tools for generating gene-specific variant information may help LSDB curation.

The standard nomenclature provides little guidance on descriptions in plain text, so multiple formats may be encountered. In addition, the standard nomenclature was not always used leading to variant descriptions in which, for example, the symbol '>' for substitution is replaced by '/,' '->', or '-->'. To catch these aberrant variant descriptions, the EBNFs presented here could easily be modified to develop parsers for text mining tools. This would not solve the problem of reference sequence identification from the Materials and Methods section or table legends. A multi-step approach including pre-processing by a lexical analyzer [24] to identify the reference sequence accession number(s) and subsequently connect that to variant descriptions identified by the Mutalyzer 2 Syntax Checker might be feasible. The Mutalyzer 2 sequence variant nomenclature checker could then be used with the format <Reference sequence.version_number>:<variant_description> to ground identified variants on a specific reference sequence accession number. Validation of the sequence variant mining results could then be carried out using the new LOVD query service [8]. Although the approaches described here may help to improve the extraction of variant descriptions from literature, authors can make a significant contribution by using the standard nomenclature, and journal editors and reviewers by enforcing its use. Obviously, listing variant descriptions in the format <Reference sequence.version_number>:<variant_description> in manuscripts or in associated computer readable files would solve many of the problems currently experienced by the text mining community.

### Conclusions

We have developed formal descriptions of the standard human sequence variant nomenclature, which can be easily modified and extended after nomenclature updates. The EBNFs can be used in combination with specific software (e.g. Pyparsing) to generate an automatic nomenclature parser. The freely accessible implementation of such a nomenclature parser, the Syntax Checker, can be used separately or as part of the Mutalyzer 2 Name Checker [22].

The rules in the EBNFs can be extended to accommodate the needs of specific disciplines, including those working with other species. We anticipate that the grammars presented here will help to close this gap by supporting the development of tools which also recognize variant descriptions at the level of DNA and RNA.

## Authors' contributions

JL, AB and PT primarily wrote the manuscript. AB created the initial grammars. PT designed HGVS nomenclature extensions, which were discussed with JL and JdD. JL included all proposed changes in the latest version of the grammar and implemented it in the Mutalyzer parser. PT and JdD supervised the project. PT finalized the manuscript. All authors read and approved the final manuscript. The authors declare that they have no competing interests.

## Supplementary Material

Additional file 1 **DNA and RNA variant nomenclature EBNF v.2.0.0.** This file contains the Extended Backus-Naur Form of the human standard DNA and RNA variant nomenclature v.2.0 used by the parser of Mutalyzer 2. Format: PDF. Click here for file

Additional file 2 **Protein variant nomenclature EBNF v.2.0.0.** This file contains the Extended Backus-Naur Form of the human standard protein variant nomenclature v.2.0. Format: PDF. Click here for file
