# Supplementary material for: A formalized description of the standard human variant nomenclature in Extended Backus-Naur Form
Source: BMC Bioinformatics. 2011 Jul 5;12(Suppl 4):S5. doi: 10.1186/1471-2105-12-S4-S5 (PMC3194197; doi:10.1186/1471-2105-12-S4-S5)
Supplement: Additional file 1 — DNA and RNA variant nomenclature EBNF v.2.0.0. This file contains the Extended Backus-Naur Form of the human standard DNA and RNA variant nomenclature v.2.0 used by the parser of Mutalyzer 2. Format: PDF. [file 1471-2105-12-S4-S5-S1.pdf]

A formalized description of the standard human variant nomenclature in Extended Backus-Naur Form Additional File 1 – DNA and RNA variant nomenclature EBNF

Jeroen F.J. Laros[1], André Blavier[2], Johan T. den Dunnen[1], Peter E. M. Taschner[1§]

[1]Department of Human Genetics, Center for Human and Clinical Genetics, Leiden University Medical Center, Leiden, Nederland

[2]Interactive Biosoftware, Rouen, France

Email: Jeroen Laros - j.f.j.laros@lumc.nl; André Blavier - ablavier@interactive-biosoftware.com; Johan den Dunnen - ddunnen@humgen.nl; Peter Taschner[§] - P.Taschner@lumc.nl

[§]Corresponding author

Extended Backus-Naur Form of DNA and RNA variant nomenclature [a]

Basic lexemes			
Nt	→	**‘a’** | **‘c’** | **‘g’** | **‘t’** | **‘u’** | **‘A’** | **‘C’** | **‘G’** | **‘T’** | **‘U’**	
NtString	→	Nt$^+$	
name	→	([a–z] | [A–Z] | [0–9])$^+$	e.g. MLH1
Number	→	([0–9])$^+$	e.g. 123

Top-level Rule

Var → SingleVar | MultiVar | MultiTranscriptVar | UnkEffectVar | NoRNAVar | SplicingVar

Locations

Loc → PtLoc | RangeLoc

Offset → (‘+’ | ‘**-**’) (‘**u**’ | ‘**d**’)? (Number | ‘**?**’)

RealPtLoc → ((‘**-**’ | ‘*’)? Number Offset?) | ‘**?**’ e.g. -200+15

IVSLoc [b] → ‘**IVS**’ Number (‘+’ | ‘**-**’) Number

PtLoc → IVSLoc | RealPtLoc

RealExtent → PtLoc ‘**_**’ (‘**o**’? (RefSeqAcc | GeneSymbol) ‘**:**’)? PtLoc

EXLoc [b] → ‘**EX**’ Number (‘**-**’ Number)?

Extent → RealExtent | EXLoc

RangeLoc → Extent e.g. 301_oXYZ:233+17
| ‘(‘ Extent ‘)’ e.g. Uncertain location

FarLoc → (RefSeqAcc | GeneSymbol) (‘**:**’ RefType? Extent)?

ChromBand → (‘**p**’ | ‘**q**’) Number ‘**.**’ Number

ChromCoords → ‘(’ Chrom ‘**;**’ Chrom ‘)’ ‘(’ ChromBand ‘**;**’ ChromBand ‘)’

Reference sequences

Ref → ((RefSeqAcc | GeneSymbol) ‘**:**’)? RefType?

RefType → (‘**c**’ | ‘**g**’ | ‘**m**’ | ‘**n**’ | ‘**r**’) ‘.’

RefSeqAcc → GenBankRef | LRG

GenBankRef → (GI | AccNo) (‘(’ GeneSymbol ‘)’)?

GI → (‘**GI**’ | ‘**GI:**’)? Number

AccNo → ‘**ish.**’? ([a–Z] Number ‘**_**’)$^+$ Version?

Version → ‘**.**’ Number

GeneSymbol → ‘**(**’ name (TransVar | ProtIso)? ‘**)**’ e.g. MLH1_v1

TransVar [c] → ‘**_v**’ Number

ProtIso [c] → ‘**_i**’ Number

LRGTranscriptID [d] → ‘**t**’ ([0-9])$^+$ e.g. LRG_1t1

LRGProteinID [d] → ‘**p**’ ([0-9])$^+$

LRG [d] → ‘**LRG**’ ([0-9])$^+$ (‘**_**’ (LRGTranscriptID | LRG-ProteinID))?

Chrom → name

Single Variations

Subst → PtLoc Nt ‘>’ Nt e.g. 123A>G

Del → Loc ‘**del**’ (Nt$^+$ | Number)? e.g. 456_458del3

Dup → Loc ‘**dup**’ (Nt$^+$ | Number)? Nest? e.g. 78_79dupCG

AbrSSR → PtLoc Nt$^+$ ‘**(**’ Number ‘**_**’ Number ‘**)**’ e.g. 7(TG)3_6

VarSSR → (PtLoc Nt$^+$ ‘[’ Number ‘]’) | (RangeLoc ‘**[**’ Number ‘**]**’) | AbrSSR

Ins → RangeLoc ‘**ins**’ (Nt$^+$ | Number | RangeLoc | FarLoc) Nest? e.g. 76_77insT

Indel → RangeLoc ‘**del**’ (Nt$^+$ | Number)? ‘**ins**’ (Nt$^+$ | Number | RangeLoc | FarLoc) Nest?

e.g. 12_15delGGACinsTA

Inv → RangeLoc ‘**inv**’ (Nt$^+$ | Number)? Nest? e.g. 77_80inv4

Conv → RangeLoc ‘**con**’ FarLoc Nest?

		e.g. c.15_355conNM_004006.1:c.15_355
TransLoc	→	‘t’ ChromCoords ‘(’ FarLoc ‘)’
		e.g. t(X;4)(p21.2;q35)(c.301-148_301-147)
RawVar	→	Subst | Del | Dup | VarSSR | Ins | Indel | Inv | Conv
SingleVar	→	Ref RawVar | TransLoc
ExtendedRawVar	→	RawVar | ‘=’ | ‘?’
UnkEffectVar	→	Ref (‘(=)’ | ‘?’)
SplicingVar	→	Ref (‘spl?’ | ‘(spl?)’)
NoRNAVar	→	Ref ‘0’ ‘?’?

Multiple Variations

CAlleleVarSet	→	ExtendedRawVar (‘;’ ExtendedRawVar)*
UAlleleVarSet	→	(CAlleleVarSet | (‘(’ CAlleleVarSet ‘)’)) ‘?’?
SimpleAlleleVarSet	→	(‘[’ UAlleleVarSet ‘]’) | ExtendedRawVar
MosaicSet	→	(‘[’ SimpleAlleleVarSet (‘/’ SimpleAlleleVarSet)* ‘]’) | SimpleAllele-VarSet
ChimeronSet	→	(‘[’ MosaicSet (‘//’ MosaicSet)* ‘]’) | Mosaic-Set
SingleAlleleVarSet	→	(‘[’ ChimeronSet ((‘;’ | ‘^’) ChimeronSet)* (‘(;)’ ChimeronSet)* ‘]’) | ChimeronSet
SingleAlleleVars	→	Ref SingleAlleleVarSet e.g. c.[76C>T; 83G>C]
MultiAlleleVars	→	Ref SingleAlleleVarSet (‘;’ Ref? SingleAlleleVarSet)$^{+}$ e.g. c.[76C>T];[76C>T]
MultiVar	→	SingleAlleleVars | MultiAlleleVars

MultiTranscriptVar → Ref ‘**[**‘ ExtendedRawVar (‘**;**’ ExtendedRawVar)* (‘**,**’ ExtendedRawVar (‘**;**’ ExtendedRawVar)*)$^+$ ‘**]**’

e.g. r.[=, 88_89ins88+1_88+10; 88+2t>c]

Nest [c] → ‘**{**’ SimpleAlleleVarSet ‘**}**’

[a] The EBNF notation is explained in the Methods section. The top level rule is the general entry point of the grammar. Basic lexemes are rules describing the characters allowed for specification of nucleotides (Nt), names and numbers. Locations are rules describing positions of variants relative to the reference sequence types used. The actual characters and symbols seen in variant descriptions, the terminal symbols, are in **bold.** See Table 1 for new symbols and [2] for a full list of symbols and their use.

[b] Deviation rules for old HGVS description formats (only used for conversion to current HGVS nomenclature)

[c] Rules specific for HGVS Extension (pending formal HGVS approval). These include transcript variant, protein isoform and nested variant descriptions [5]

[d] Rules specific for LRG (Locus Reference Genomic, http://www.lrg-sequence.org/)[18]
