# Supplementary material for: A formalized description of the standard human variant nomenclature in Extended Backus-Naur Form
Source: BMC Bioinformatics. 2011 Jul 5;12(Suppl 4):S5. doi: 10.1186/1471-2105-12-S4-S5 (PMC3194197; doi:10.1186/1471-2105-12-S4-S5)
Supplement: Additional file 2 — Protein variant nomenclature EBNF v.2.0.0. This file contains the Extended Backus-Naur Form of the human standard protein variant nomenclature v.2.0. Format: PDF. [file 1471-2105-12-S4-S5-S2.pdf]

A formalized description of the standard human variant nomenclature in Extended Backus-Naur Form

Additional File 2 – Protein variant nomenclature EBNF

Jeroen F.J. Laros[1], André Blavier[2], Johan T. den Dunnen[1], Peter E. M. Taschner[1§]

[1]Department of Human Genetics, Center for Human and Clinical Genetics, Leiden University Medical Center, Leiden, Nederland

[2]Interactive Biosoftware, Rouen, France

Email: Jeroen Laros - j.f.j.laros@lumc.nl; André Blavier - ablavier@interactive-biosoftware.com; Johan den Dunnen - ddunnen@humgen.nl; Peter Taschner[§] - P.Taschner@lumc.nl

[§]Corresponding author

Extended Backus-Naur Form of protein variant nomenclature v.2.0.0 [a]

Basic lexemes		
AA	→	AA1 | AA3 | ‘**X**’
AA1	→	‘**A**’ | ‘**R**’ | ‘**N**’ | ‘**D**’ | ‘**C**’ | ‘**Q**’ | ‘**E**’ | ‘**G**’ | ‘**H**’ | ‘**I**’ | ‘**L**’ | ‘**K**’ | ‘**M**' | ‘**F**' | ‘**P**’ | ‘**S**’ | ‘**T**’ | ‘**W**’ | ‘**Y**’ | ‘**V**’
AA3	→	‘**Ala**’ | ‘**Arg**’ | ‘**Asn**’ | ‘**Asp**’ | ‘**Cys**’ | ‘**Gln**’ | ‘**Glu**’ | ‘**Gly**’ | ‘**His**’ | ‘**Ile**’ | ‘**Leu**’ | ‘**Lys**’ | ‘**Met**’ | ‘**Phe**’ | ‘**Pro**’ | ‘**Ser**’ | ‘**Thr**’ | ‘**Trp**’ | ‘**Tyr**’ | ‘**Val**’
Number	→	$[0-9]^{+}$

Name	→	[a-zA-Z0-9_]$^+$
Top-level Rule		
ProteinVar	→	SingleVar | MultiVar
Locations		
AALoc	→	AAPtLoc | AARange
AAPtLoc	→	AA PtLoc
PtLoc	→	(‘**-**’ | ‘*****’)? Number<br>| Number (‘**+**’ | **‘-**’) Number
AARange	→	Extent<br>| ‘(’ Extent ‘)’
Extent	→	AAPtLoc ‘**_**’ AAPtLoc
Reference sequences		
Ref	→	(Name ‘**:**’)? ‘**p.**’
Single Variations		
SingleVar	→	Ref RawVar
RawVar	→	Subst | Del | Dup | VarSSR<br>| Ins | Indel | FrameShift<br>| ‘**=**’ | ‘**?**’ | ‘**0**’ | ‘**0?**’
Subst	→	AAPtLoc AA (‘**extX**’ ‘*****’? Number)?<br>| (‘**Met1**’ | ‘**M1**’) (‘**?**’ | ‘**ext**’ ‘**-**’ Number)
Del	→	AALoc ‘**del**’
Dup	→	AALoc ‘**dup**’
VarSSR	→	AALoc ‘**(**’ Number ‘**_**’ Number ‘**)**’
Ins	→	AARange ‘**ins**’ (AA$^+$ | Number)
Indel	→	AALoc ‘**delins**’ (AA$^+$ | Number)
FrameShift	→	ShortFS | LongFS
ShortFS	→	AAPtLoc ‘**fs**’
LongFS	→	AAPtLoc AA ‘**fs**’ ‘**X**’ Number
Multiple Variations		
MultiVar	→	SingleAlleleVars<br>| MultiAlleleVars<br>| UnkAlleleVars
SingleAlleleVars	→	Ref SingleAlleleVarSet

SingleAlleleVarSet	→	‘**[**’ RawVar<br>(‘**;**’ RawVar)$^+$ | (‘**,**’ RawVar)$^+$<br>‘**]**’
MultiAlleleVars	→	Ref SingleAlleleVarSet ‘;’<br>Ref? SingleAlleleVarSet
UnkAlleleVars	→	Ref ‘[’ RawVar ‘**(;)**’ RawVar ‘]’

[a] Please note that although the names of the production rules in the two EBNFs may be similar, the rules themselves are different. Terminal symbols are in **bold**. See [2] for a full list of symbols and their use.
